# Enhancing the Cellular Robustness of Cyanobacteria to Improve the Stability and Efficiency of Bio-Photovoltaics

**DOI:** 10.3390/life15020299

**Published:** 2025-02-14

**Authors:** Xiangyi Yuan, Xuejing Xu, Xuemin Gao, Xiangxiao Liu, Bo Liang, Guodong Luan, Xuefeng Lu

**Affiliations:** 1 Energy-Rich Compounds Production by Photosynthetic Carbon Fixation Research Center, Shandong Key Lab of Applied Mycology, College of Life Sciences, Qingdao Agricultural University, Qingdao 266109, China; yuanxiangyi@qibebt.ac.cn (X.Y.); gxm20000209@163.com (X.G.); liuxiangxiao@qibebt.ac.cn (X.L.); 2 Key Laboratory of Photoelectric Conversion and Utilization of Solar Energy, Qingdao Institute of Bioenergy and Bioprocess Technology, Chinese Academy of Sciences, No. 189 Songling Road, Qingdao 266101, China; xuxj@qibebt.ac.cn (X.X.); lvxf@qibebt.ac.cn (X.L.); 3 Qingdao New Energy Shandong Laboratory, Qingdao 266101, China; 4 College of Life Science, University of Chinese Academy of Sciences, Beijing 100049, China

**Keywords:** cyanobacteria, bio-photovoltaics, FoF1-ATP synthase, cellular robustness

## Abstract

Solar photovoltaic technology has consistently been regarded as a crucial direction for the development of clean energy systems in the future. Bio-photovoltaics (BPV), an emerging solar energy utilization technology, is mainly based on the photosynthesis process of photoautotrophic organisms to convert solar energy into electrical energy and output a photocurrent via extracellular electron transfer. As the fundamental unit of the bio-photovoltaic system, the stability of photosynthetic microorganisms under fluctuating and stressful light and heat conditions is likely to have a significant influence on the efficiency of bio-photovoltaic devices. However, this aspect has often been overlooked in previous bio-photovoltaics research. This study took an important cyanobacteria chassis strain, *Synechococ elongatus* PCC 7942, as the model organism and explored the impact of physiological robustness optimization on its performance as a bio-photovoltaic functional unit. In this work, two types of BPV systems, namely the suspension mode and the biofilm attachment mode, were assembled to evaluate the electricity-generating activity of *Synechococcus* cells. Overall, the latter demonstrated a remarkable photoelectric output performance. When its light and temperature tolerance was enhanced through FoF1-ATP synthase engineering, the optimized *Synechococcus* strain exhibited stronger photosynthetic physiology and photoelectric output activity. Under the condition of a light intensity of 2400 μmol photons/m^2^/s, the maximum photocurrent output of the *Synechococcus*-based BPV device was increased significantly by 41% over the system based on the wild-type control strain. The results of this study provided a new perspective for the future development and optimization of bio-photovoltaics.

## 1. Introduction

Solar energy stands as a vital renewable energy source, and solar photovoltaic power generation is regarded as a key element within the future clean energy supply system. The progress of traditional photovoltaic technology is rooted in the photoelectric effect detected in various semiconductor materials. This has led to a conversion efficiency surpassing 20%, and the technology has been successfully incorporated into extensive industrial applications [1,2,3]. Bio-photovoltaics (hereafter termed as BPV for short) is an emerging and developing photovoltaic conversion technology. Its core concept is centered around using the photosynthetic systems in microorganisms or plant cells for photovoltaic conversion. Through extracellular electron transfer, it generates a current output [4,5,6,7]. The photosynthetic cells integrated in BPV systems have the abilities to carry out self-replication and self-repair. These characteristics endow BPV devices with potential advantages such as being environmentally friendly and durable. As a result, BPV is highly suitable for fulfilling the power requirements in certain special off-grid scenarios [8,9,10].

Cyanobacteria, renowned as one of the most representative photosynthetic microorganisms with the highest photosynthetic efficiency, are extensively employed in the construction of BPV systems. However, the weak extracellular electron transfer (EET) capacity of cyanobacteria has emerged as a key bottleneck constraining the effectiveness of BPV systems [11,12]. To surmount the barrier effects of cyanobacterial cell envelopes, numerous strategies have been implemented, including genetic modification, the addition of electron mediators, and the construction of biomaterial hybrids. These approaches have significantly enhanced the output power of cyanobacterial BPV systems [13,14,15,16]. In the design and optimization of BPV systems, apart from EET activity, the efficiency and stability of the cyanobacterial photosynthetic system, which is another factor potentially exerting a significant impact on the performance of bio-photovoltaics, are frequently overlooked. In the practical application and operation of BPV, the conditions cyanobacteria experience during photosynthesis differs from the stable laboratory settings. Instead, they are more likely to encounter various fluctuating and even stressful environmental parameters. For example, the intense light in the outdoor environment that exceeds the adaptability of cyanobacteria can not only fail to effectively support photoelectric conversion, but also cause damage to the physiology and metabolism of the cells. Moreover, the high temperature stress that often accompanies the intense light will further exacerbate the damage to the cells. Therefore, strengthening the cellular robustness of cyanobacteria, which serve as the fundamental units of the bio-photovoltaic system, is conducive to ensuring the performance of the bio-photovoltaic system under complex and harsh environments. This, in turn, improves the efficiency and stability of photoelectric conversion.

In this work, we employed *Synechococcus elongatus* PCC 7942 as a model to explore the feasibility of enhancing the efficiency and stability of cyanobacterial BPV systems by improving the physiological robustness of cyanobacteria cells. Our primary research hypothesis is that enhancing the high light and temperature tolerance of cyanobacteria through FoF1-ATP synthase engineering will lead to improved photosynthetic physiology and photoelectric output activity in cyanobacteria when used as functional units in BPV systems. Specifically, we hypothesize that the modified *Synechococcus* strain with enhanced tolerance will exhibit a higher photocurrent output compared to the wild-type strain under high light and high temperature conditions. Based on the highly tolerant strain previously obtained through FoF1-ATP synthase modifications [17], we evaluated the performance of the BPV system with it as the functional unit under high-temperature and high-light conditions and compared it with the system based on the wild-type control strain. The results demonstrated that the output of the robust strain-derived BPV system was significantly enhanced in both the suspension mode and the biofilm attachment mode, and in the subsequent photophysiological and photochemical analyses, the advantage of the highly tolerant strain in photocurrent output was also corroborated by its superiority in a series of parameters. These results validate the efficacy and potential of developing and optimizing efficient and stable BPV systems through enhancing the cellular robustness of cyanobacterial cells serving as functional units.

## 2. Materials and Methods

### 2.1. Scheme of the Described Studies

To investigate the possible impact of the stability of photosynthetic microorganisms as the basic unit of a BPV system under fluctuating and laser-stress thermal conditions on the efficiency of a BPV installation. This study took an important cyanobacteria chassis strain, *Synechococcus elongatus* PCC 7942, as the model organism and explored the impact of physiological robustness optimization on its performance as a BPV functional unit. When the high light and high temperature tolerance was enhanced through FoF1-ATP synthase engineering, the optimized *Synechococcus* strain exhibited a stronger photosynthetic physiology. Subsequently, two distinct BPV devices were constructed using a highly tolerant cyanobacteria strain and a wild-type cyanobacteria strain. This study investigates the impact of the inherent resistance of photosynthetic microorganisms on the output of these devices by comparing their differences in output efficiency and stability.

### 2.2. Strains and Cultivation

*Synechococcus elongatus* PCC 7942 and a derived robust strain HL7942 with a modified alpha subunit of the FoF1-ATP synthase (the amino acid at position 252 is replaced by tyrosine instead of cysteine) were utilized to construct the BPV system [17]. *Synechococcus* strains were cultivated in BG11 medium with the following compositions (per liter): 1.5 g NaNO_3_, 0.047 g K_2_HPO_4_·3H_2_O, 0.075 g MgSO_4_·7H_2_O, 0.027 g CaCl_2_, 0.02 g Na_2_CO_3_, 0.006 g citric acid, 0.006 g ammonium ferric citrate, 0.001 g EDTA disodium salt dihydrate, 1 mL trace element stock solution. The trace element stock solution contained (per liter) 2.86 g H_3_BO_3_, 1.81 g MnCl_2_·4H_2_O, 0.222 g ZnSO_4_·7H_2_O, 0.079 g CuSO_4_·5H_2_O, 0.391 g Na_2_MoO_4_·2H_2_O and 0.04 g CoCl_2_·6H_2_O. The cultivation was conducted at 30 °C in an illumination shaker with agitation set to 150 rpm under a light intensity of 50 μmol photons/m^2^/s.

### 2.3. Photophysiological and Photochemical Characterizations

The photochemical activities of photosystem II (PS II) and photosystem I (PS I) of the cyanobacteria were determined by measuring chlorophyll fluorescence parameters using a Dual-PAM-100 fluorometer (Walz, Free State of Bavaria, Germany). To better explain the biological phenotypes of algal strains in the BPV installations, this study adopts the conventional analytical standard of optical density (OD) in the field of BPV as the benchmark for determining and analyzing photosynthetic parameters. A 1.5 mL *Synechococcus* culture broth was sampled when the optical density at a light wavelength of 730 nm reached 0.6 and was added to a quartz cuvette with four smooth sides for measurement. The slow induced curve and light response curve were monitored to obtain the chlorophyll fluorescence kinetic parameters.

### 2.4. Assembly of BPV Systems

The electrochemical apparatus employed for photocurrent measurement consisted of a three-electrode system, which included a working electrode, a counter electrode (a platinum sheet electrode) and a reference electrode (Ag/AgCl electrode filled with a saturated AgCl solution). Indium Tin Oxide (ITO)-conductive glass was utilized as the substrate for the electrode, and a cyanobacterial biofilm was deposited to on it to generate a working electrode. BG11 medium, devoid of ferric ammonium citrate, was employed as the electrolyte to avoid the negative effects of the redox reactions of iron atoms on the photocurrent.

(1) Cyanobacteria biofilm attachment pattern BPV system: to prepare the working electrode, approximately 3 × 10^8^ *Synechococcus* cells were collected, washed and resuspended in 100 μL electrolyte. Subsequently, the cell suspension was dropped onto an ITO-conductive glass and left to form a stable biofilm at room temperature. The ITO conductive glass was installed into the bio-electrochemical system.
(2) Cyanobacteria cells suspension pattern BPV system: Photocurrent measurement was conducted by using a CHI1030C potentiostat (CH Instruments, Shanghai, China). The three electrodes in the bio-electrochemical system were electrically connected with the potentiostat. Photocurrent measurements were performed using chronoamperometry.

## 3. Results

### 3.1. Setup of the BPV Systems

**Figure 1:**
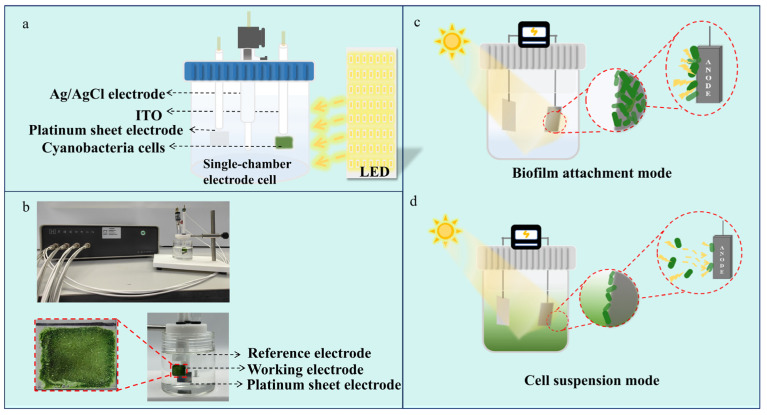
Setup of the BPV system. Design (**a**) and actual pictures (**b**) of the media-free single-chamber BPV system. As the basic functional units, cyanobacteria cells are integrated into the system in the mode of biofilm (**c**) or suspension (**d**).

Multiple cyanobacteria species, including *Synechocystis* sp. PCC 6803 [18,19,20,21], *Synechococcus* sp. [22,23], *Nostoc punctiforme* [24,25], *Microcystis aeruginosa* [26], *Leptolyngbya* sp. [27] and *Spirulina platensis* [28,29,30,31], etc., have been utilized in BPV. Although there are significant differences in photosynthetic efficiency and stability among different species or strains, when they are used in the construction of BPV systems, the differences in their photoelectric output performances may be caused by multiple factors, such as extracellular electron transfer activity, cell morphology and the strength of cell–electrode interface affinity. Thus, it would be difficult to determine the contribution of their cellular physiological robustness. To minimize the influence of other cellular physiological and metabolic factors and thus better assess the contribution brought about by the optimization of cellular robustness, we designed a strategy that is based on minimizing genomic modifications, enhancing the physiological robustness of a single strain and then evaluating its impact on the photoelectric output performance when used in BPV construction. In practical operation, we took a typical cyanobacterial strain, *Synechococcus elongatus* PCC 7942 (termed as WT7942 for short), as the model and selected HL7942, a derived mutant strain from it, which has only a single base difference in the genome (the amino acid at position 252 of FoF1-ATP synthase alpha subunit is replaced by tyrosine instead of cysteine) compared with WT7942 but has a significantly improved resistance to high temperatures and high light intensity. We compared the efficiency of bio-photovoltaics constructed with these two strains (WT7942 and HL7942) as units, hoping to reveal the influence of the stress resistance of photosynthetic cells. A medium-free single-chamber bio-photovoltaic system was developed, aiming to evaluate and compare the light response outputs of different cyanobacteria strains while reducing the background output noise. The cyanobacteria cells were integrated into this system as basic functional units in both the suspension and the biofilm attachment mode (Figure 1).

### 3.2. Photoelectrical Response of the Synechococcus Cells to Gradient Light Intensities

**Figure 2:**
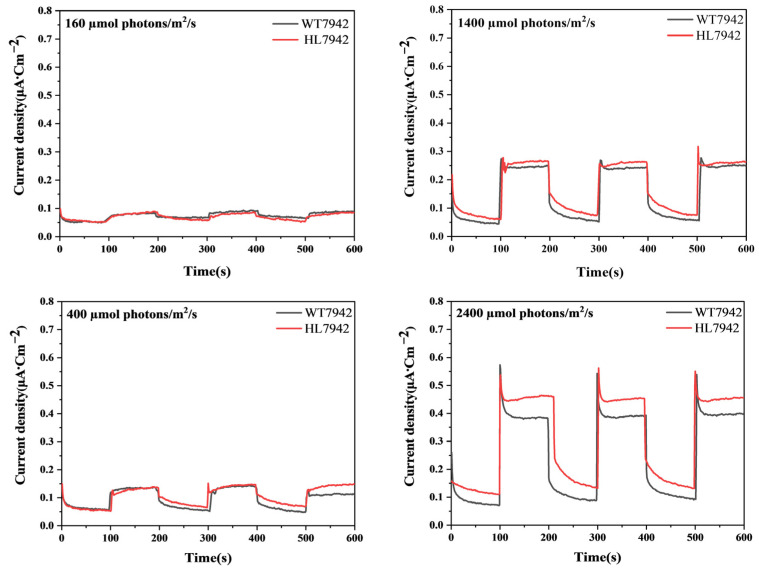
The photocurrent output response of the *Synechococcus* biofilm attachment pattern BPV system to gradient light intensities. The BPV was irradiated with LED light in the intensity range from 160 μmol photons/m^2^/s to 2400 μmol photons/m^2^/s in a light–dark cycle of 100 s/100 s, and the output current was measured using chronoamperometry.

Based on the biofilm attachment mode, we initially carried out a preliminary characterization of the current output of the BPV systems constructed using WT7942 and HL7942 under varying gradient light intensities. As shown in Figure 2, in the On-Switch mode (where each round consists of 100 s of illumination followed by 100 s of the dark phase), as the light intensity gradually increased from 160 μmol photons/m^2^/s to 2400 μmol photons/m^2^/s, both the WT7942 and HL7942 cells attached to the electrode showed a tendency for the current output to rise in accordance with the increase in light intensity. Under relatively low light intensities, specifically within the range tested, there was no significant difference in the current output between the BPV system constructed with WT7942 and that with HL7942. However, when the light intensity reached 2400, the average photocurrent output of the mutant strain HL7942 was 0.414 μA/cm^2^, representing a significant improvement compared to the wild-type photocurrent output of 0.396 μA/cm^2^, and a notable difference emerged. The observed experimental phenomenon can be attributed to the fact that the physiological activity of both cyanobacteria strains remained unaffected across a range of light intensities. Consequently, the photocurrent output levels of the devices were comparable and exhibited minimal variation when the energy input to the system was consistent. Upon further increasing the light intensity, the physiological activity of WT7942 was significantly inhibited, while the physiological activity of the mutant strain remained unchanged. Consequently, significant differences in photocurrent output were observed when the system was subjected to the same high-energy input. In multiple detection rounds under this high light intensity, the output of the BPV system using HL7942 was consistently and stably higher than that of the system using WT7942. It has been generally observed in previous research that, when the light intensity exceeds 1000 μmol photons/m^2^/s, the photosynthetic growth of WT7942 is severely inhibited. In contrast, HL7942 demonstrated a better adaptability under such high-light-intensity conditions [32]. These results collectively suggest that, compared to the photosynthesis activity, *Synechococcus* cells, when utilized as functional units in BPV systems, exhibit the ability to support photoelectric current output within a broader range of light intensities. This finding implies that the enhanced adaptability of HL7942 cells, as evidenced by their higher current output under high light intensity, may have implications for the optimization and application of BPV systems in various light environments.

### 3.3. Photoelectrical Response of the Synechococcus Cells Toward Long-Term Strong Illuminations

**Figure 3:**
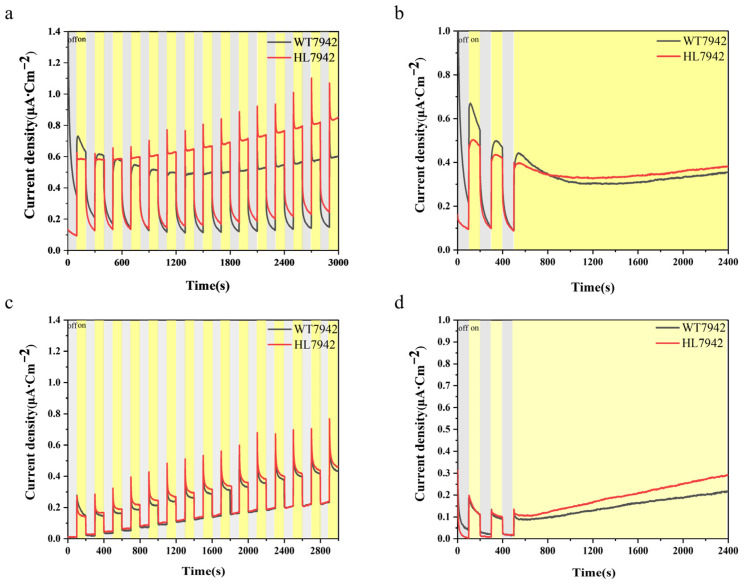
The photocurrent output response of the BPV system to long-term strong illuminations. The *Synechococcus* cells were integrated into the BPV system in the form of a biofilm attachment pattern (**a**,**b**) or a suspension pattern (**c**,**d**). The BPV was irradiated with LED light with the intensity of 2400 μmol photons/m^2^/s in a light–dark cycle of 100 s/100 s (**a**,**c**) or in continuous mode (**b**,**d**), and the output current was measured using chronoamperometry.

Under the light intensity of 2400 μmol photons/m^2^/s, HL7942 demonstrated a superior photoelectric current output capability compared to WT7942. Consequently, we proceeded to conduct systematic measurements and comparisons on the performances of WT7942 and HL7942 cells in both the suspension and biofilm attachment patterns under long-term illuminations and periodic illuminations. As shown in Figure 3a, during multiple light–dark cycles, the average photoelectric current output of the HL7942 biofilm attachment pattern BPV system reached 0.845 μA/cm^2^. This value was notably 41% higher than the output level (0.599 μA/cm^2^) of the control system constructed using WT7942. When the BPV systems were evaluated in the mode of long-term continual illumination (Figure 3b), the average photoelectric current outputs of the two systems decreased significantly. The observed decrease in the photocurrent output density during the assay was attributed to the physiological stress experienced by the wild-type cyanobacteria strain at a light intensity of 2400 μmol photons/m^2^/s. In contrast, HL7942 exhibited better tolerance, resulting in a relatively stable photocurrent output level, which indicated a crossover in the output results. However, even in this challenging condition, the HL7942 system still exhibited its advantages, with an average photocurrent output of 0.342 μA/cm^2^ compared to 0.317 μA/cm^2^ of the WT7942 system. It is also noteworthy that, after the measurement, when the indium tin oxide (ITO)-conductive glass was disassembled, the *Synechococcus* cells on the electrode surface all showed relatively severe damage, presenting a faded and bleached appearance. Intriguingly, the bleaching phenomenon of HL7942 cells was significantly less pronounced than that of the wild-type control, which further confirmed the stronger robustness of this cyanobacteria strain. Furthermore, the performances of the two strains as BPV functional units were also evaluated in the suspension pattern BPV. As shown in Figure 3c,d, in both the light–dark and long-term illumination modes, BPV systems utilizing HL7942 showed 10–30% higher photocurrent outputs than the WT7942 system. During the measurement process, the electrodes are influenced by a transient polarization phenomenon resulting from rapid fluctuations in circuit current. The degree of polarization varies across different electrodes and measurement methods, leading to distinct polarization peaks (Figure 3a,c). The data presented in this manuscript have not been past-polarized to show different output patterns. These results collectively highlight the superior performance and robustness of HL7942 in BPV applications across different illumination patterns and conditions.

### 3.4. Photophysiological Evaluation of the Synechococcus Cells for BPV Development

**Figure 4:**
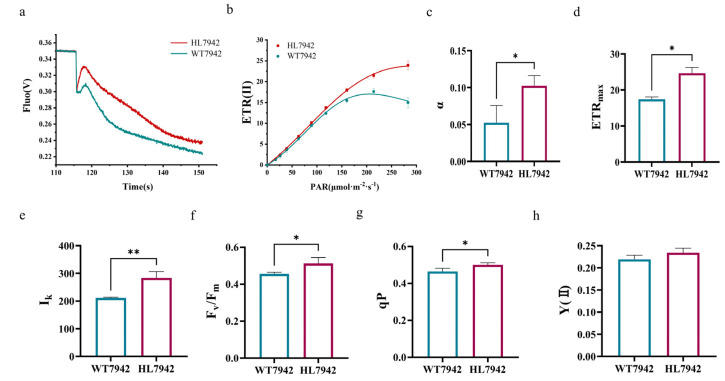
Photophysiological parameters of the HL7942 and WT7942 cells. In the photosynthetic parameter determination measurements, the red curve represents the HL7942 experimental data and the dark green curve represents WT7942. (**a**) Measurement of post−illumination fluorescence: CEF around PS I; (**b**) RLC: reflects the present photosynthetic state of the sample and also reflects the potential photosynthetic activity of the sample at different ambient light intensities; (**c**) α is the initial slope of the fast light curve, reflecting photosynthetic organs’ efficiency of light energy utilization (*p* value = 0.0255); (**d**) ETR_max_ is the fitted potential maximum relative electron transfer efficiency (*p* value = 0.0102); (**e**) IK reflects the proportion of energy absorbed by PS II (*p* value = 0.0032); (**f**) Fv/Fm: maximum light energy conversion efficiency of PS II (*p* value = 0.0158). (**g**) qP reflects the proportion of energy absorbed by PS II that is used to carry out photochemical reactions (*p* value = 0.0357); (**h**) Y(II) shows the actual light energy conversion efficiency of PS II. The photosynthetic activity tests all showed that HL7942 was stronger than WT7942. The method of significance analysis used here was *t*-tests, where a *p* value < 0.05 signifies * and a *p* value < 0.01 signifies **.

In different BPV modes, namely biofilm attachment pattern and suspension pattern, as well as under various light illumination modes such as long-term illuminations and periodic illuminations, the more robust strain HL7942 consistently outperformed the wild-type control WT7942 in terms of photoelectric output capabilities. To delve deeper into the underlying reasons for this superiority in photoelectric activity, we meticulously characterized the photophysiological parameters of the two types of *Synechococcus* cells, as depicted in Figure 4.

The observed decrease in the photocurrent output density during the assay was attributed to physiological stress experienced by the wild-type cyanobacteria strain at a light intensity of 2400 μmol photons/m^2^/s. In contrast, HL7942 exhibited better tolerance, resulting in a relatively stable photocurrent output level, which indicated a crossover in the output results.

Post-illumination fluorescence, precisely quantified using the Dual-PAM-100 software and instrument, displayed a significantly higher intensity for HL7942 (Figure 4a). This observation implies a more efficient cyclic electron flow around PS I, which presumably plays a crucial role in enhancing the electron supply, which is essential for photocurrent generation. The rapid light curve (RLC), derived through the modulated chlorophyll fluorescence technique, unveiled that HL7942 boasted higher values in several key parameters. The initial slope α, which reflects the light energy utilization efficiency (Figure 4c), was notably greater in HL7942. This higher α value empowers the strain to make better use of weak light, thereby ensuring a stable electron production even under low−light conditions. Furthermore, following a 15 min dark treatment of the cells, the ETR_max_, representing the fitted potential maximum relative electron transfer efficiency (Figure 4d), was also higher in HL7942. A greater ETR_max_ indicates a higher capacity for electron transfer, directly contributing to an increased photocurrent. Moreover, the IK parameter, which reflects the strong light tolerance (Figure 4e), was superior in HL7942. This means that HL7942 exhibits better resistance to intense light, enabling it to maintain its photosynthetic performance and electron flow across a wide range of light intensities. Furthermore, the fluorescence induction parameters, obtained after dark treatment and the continuous fluorescence-induced reaction, demonstrated that HL7942 possessed a greater light energy conversion efficiency in PS II. This was evidenced by higher values in Fv/Fm (Figure 4f), qP (reflecting the proportion of energy used for photochemical reactions, Figure 4g) and Y(II) (reflecting the actual light energy conversion efficiency, Figure 4h). These higher values suggest that HL7942 is more proficient in converting light energy into chemical energy and utilizing it for photosynthesis. This enhanced conversion and utilization ultimately lead to an increased availability of electrons for photocurrent generation.

In summary, the outstanding performance of HL7942 across various photophysiological parameters, encompassing post-illumination fluorescence, RLC parameters, and fluorescence induction parameters, comprehensively elucidates its enhanced photocurrent output in the context of bio-photovoltaics. These findings not only shed light on the underlying mechanisms but also underscore the immense potential of HL7942 in elevating the efficiency and practicality of bio-photovoltaic technologies, opening up new avenues for their development and application in the future.

## 4. Discussion

BPV technology holds great promise as a clean and renewable energy source. However, its widespread adoption is currently hindered by a low output power. Among the various methods used to boost the power output, screening and modifying photosynthetic microorganisms is a cost-effective approach [33,34,35]. But, typically, enhancing BPV output efficiency often involves introducing an exogenous extracellular electron transfer pathway into photosynthetic microorganisms, which is complex and challenging [13,36]. In this study, we adopted a different strategy. We focused on enhancing the optical collection of expanded cyanobacteria while maintaining a certain photoelectron output efficiency. To achieve this, we increased the environmental light intensity and simultaneously modified the cyanobacteria physiochemically. We used a mutant *Synechococcus* strain, HL7942 (FoF1-AtpA- C252Y), with enhanced environmental resilience for the photocurrent assays.

Comparing our results with previous studies, our approach of enhancing cellular robustness through FoF1-ATP synthase engineering offers a novel perspective, despite not achieving an order-of-magnitude increase in the current output intensity. In the field of BPV technology, most prior research has been fixated on strategies like adding electron mediators or constructing biomaterial hybrids to enhance extracellular electron transfer activity [37,38,39]. While these methods have led to some improvements in the output power of cyanobacterial BPV systems, they have overlooked a crucial aspect—the stability and robustness of photosynthetic cells. The stability of photosynthetic microorganisms under fluctuating environmental conditions, such as varying light and temperature, has long been underestimated. Our study reveals that there is significant untapped potential in this area. By enhancing the physiological robustness of cyanobacteria, we have shown that it is possible to optimize the performance of BPV systems, even without a dramatic increase in current output on a quantitative scale. This new understanding emphasizes the importance of considering the adaptability of cyanobacteria to environmental stress in future research.

Under different light intensity conditions, the HL7942 strain showed remarkable advantages. In the photoelectrical response to gradient light intensities, when the light intensity reached 2400 μmol photons/m^2^/s, the BPV system using HL7942 had a consistently higher output than that using the wild-type WT7942 strain. This indicates that HL7942 can support photoelectric current output in a broader range of light intensities. In the photophysiological evaluation, HL7942 also outperformed WT7942 in multiple parameters. For example, it had a higher post-illumination fluorescence intensity, which implies a more efficient cyclic electron flow around PS I, ensuring a better electron supply for photocurrent generation. Its higher initial slope α in the Rapid Light Curve (RLC) shows a better light energy utilization efficiency, especially in weak light conditions. And the higher ETR_max_ value indicates a greater electron transfer capacity, directly contributing to an increased photocurrent. Recently, the strategy of optimizing cellular robustness has emerged as a powerful tool in the construction of advanced photosynthetic cell factories. By enhancing the stress tolerance and metabolic stability of photosynthetic microorganisms, these cell factories have achieved significant improvements in productivity. For instance, some factories have successfully increased the production of high-value metabolites like biofuels and pharmaceuticals by engineering strains with enhanced cellular robustness [40,41]. This approach has enabled the cells to better withstand harsh environmental conditions during the production process, leading to more stable and efficient production. Our current study extends the application of this strategy to the field of bio-photovoltaics. It clearly demonstrates that when cyanobacteria are employed as photoelectric output units, optimizing cellular robustness through FoF1-ATP synthase engineering can effectively enhance the performance of BPV systems. This finding not only validates the broad-spectrum effectiveness of the cellular robustness optimization strategy but also paves the way for further innovation in the design and development of bio-photovoltaics, suggesting new directions for improving the efficiency and stability of this renewable energy technology.

In future research endeavors, it is of the utmost significance to rationally modify and optimize cyanobacteria strains in accordance with the specific stressors encountered by bio-photovoltaic systems. By bolstering the tolerance of phototrophic microorganisms to environmental conditions, the stability of the entire BPV system can be further enhanced. Moreover, the introduction of an intelligent dynamic response loop holds the key to improving the stability of photoelectric output. This loop could be designed to sense real-time environmental changes, such as fluctuations in light intensity, temperature, and nutrient availability, and then adjust the metabolic and physiological processes of the phototrophic microorganisms accordingly. In conclusion, this study provides valuable design concepts and perspectives for the construction of BPV systems in extreme environments in the future. It serves as a crucial stepping-stone for the more efficient and practical application of this promising renewable energy technology, potentially enabling BPV systems to be deployed in a wider range of scenarios, from remote off-grid areas to harsh industrial environments.

## 5. Conclusions

This study explored a novel strategy to enhance BPV performance. By modifying cyanobacteria to improve their light-collecting ability and tolerance to intense light, we increased the photocurrent output. The mutant strain HL7942 outperformed the wild-type WT7942 under strong-light conditions. Future research should focus on rational cyanobacteria strain modification based on BPV system stressors, enhancing microorganism environmental tolerance, and implementing an intelligent dynamic response loop for stable photoelectric output. In summary, this study provides valuable insights for constructing BPV systems in extreme environments, facilitating the practical and efficient application of this renewable energy technology across diverse scenarios.

## Data Availability

The data supporting the conclusions of this article will be made available by the authors on request.

## Abbreviations

BPV, bio-photovoltaics; PCC 7942, *Synechococcus elongatus* PCC 7942; WT, wild-type; PS I, photosystem I; PS II, photosystem II; ITO, Indium Tin Oxide; EET, extracellular electron transfer.
